# Supplementary material for: Occupancy estimation of wild species in a palm oil plantation using unstructured data
Source: PLoS One. 2026 Feb 2;21(2):e0328960. doi: 10.1371/journal.pone.0328960 (PMC12863681; doi:10.1371/journal.pone.0328960)
Supplement: S2 Table — Model 1 is the most comprehensive model described in the main text. The others are models in which one or more covariates have been omitted. No entry indicates that the covariate is not included in the model. (DOCX) [file pone.0328960.s002.docx]

Table S2. Overview of models. Model 1 is the most comprehensive model described in the main text. The others are models in which one or more covariates have been omitted. No entry indicates that the covariate is not included in the model.

| Model  ID | Habitat of site | Distance  to forest | Site size | List  Length | Observer | Habitat of observation |
| --- | --- | --- | --- | --- | --- | --- |
| 1 | x | x | x | x | x | x |
| 2 |  | x | x | x | x | x |
| 3 | x |  | x | x | x | x |
| 4 | x | x |  | x | x | x |
| 5 | x | x | x |  | x | x |
| 6 | x | x | x | x |  | x |
| 7 | x | x | x | x | x |  |
| 8 |  |  | x | x | x | x |
| 9 | x |  |  | x | x | x |
| 10 | x | x |  | x | x |  |
| 11 |  | x |  | x | x | x |
| 12 |  | x | x | x | x |  |
| 13 | x |  | x | x | x |  |
| 14 |  |  |  | x | x | x |
| 15 |  |  | x | x | x |  |
| 16 | x |  |  | x | x |  |
| 17 |  | x |  | x | x |  |
| 18 |  |  |  | x | x |  |
