# Supplementary material for: Occupancy estimation of wild species in a palm oil plantation using unstructured data
Source: PLoS One. 2026 Feb 2;21(2):e0328960. doi: 10.1371/journal.pone.0328960 (PMC12863681; doi:10.1371/journal.pone.0328960)
Supplement: S3 Table — See S2 Table for the meaning of model IDs. (DOCX) [file pone.0328960.s003.docx]

Table S3. Top model per species per dataset according to WAIC. See table S1 for the meaning of model IDs.

The species are arranged by class: 13 birds, 3 reptiles and 7 mammals (3 rodents and 4 monkeys).

| Species | Standard | Nondet3grp | Nondet3grp+obs1grp | Nondet3grp+obs3grp | Remove_sites1yr | Remove_sites2024 |
| --- | --- | --- | --- | --- | --- | --- |
| ***Birds*** |  |  |  |  |  |  |
| *Acridotheres javanicus* | 8 | 8 | 8 | 8 | 8 | 8 |
| *Alcedo meninting* | 1 | 1 | 1 | 1 | 1 | 1 |
| *Amaurornis phoenicurus* | 8 | 8 | 3 | 8 | 8 | 8 |
| *Anthracoceros malayanus* | 10 | 10 | 10 | 10 | 10 | 10 |
| *Centropus sinensis* | 8 | 3 | 8 | 3 | 8 | 8 |
| *Corvus enca* | 13 | 13 | 13 | 13 | 13 | 13 |
| *Egretta garzetta* | 1 | 1 | 1 | 2 | 2 | 2 |
| *Elanus caeruleus* | 12 | 12 | 12 | 7 | 12 | 7 |
| *Gracula religiosa* | 1 | 1 | 1 | 13 | 3 | 3 |
| *Halcyon smyrnensis* | 14 | 14 | 11 | 11 | 14 | 14 |
| *Hirundo tahitica* | 11 | 2 | 11 | 2 | 14 | 11 |
| *Spilopelia chinensis* | 11 | 11 | 11 | 11 | 11 | 11 |
| *Spilornis cheela* | 13 | 13 | 7 | 13 | 7 | 12 |
|  |  |  |  |  |  |  |
| ***Reptiles*** |  |  |  |  |  |  |
| *Liopeltis tricolor* | 15 | 15 | 15 | 15 | 13 | 15 |
| *Malayopython reticulatus* | 2 | 2 | 2 | 2 | 2 | 2 |
| *Varanus salvator* | 14 | 11 | 11 | 8 | 8 | 2 |
|  |  |  |  |  |  |  |
| ***Mammals (rodents)*** |  |  |  |  |  |  |
| *Callosciurus notatus* | 7 | 12 | 12 | 7 | 7 | 12 |
| *Callosciurus prevostii* | 3 | 8 | 8 | 3 | 8 | 8 |
| *Nannosciurus melanotis* | 1 | 1 | 1 | 3 | 1 | 3 |
| Species | Standard | Nondet3grp | Nondet3grp+obs1grp | Nondet3grp+obs3grp | Remove_sites1yr | Remove_sites2024 |
|  |  |  |  |  |  |  |
| ***Mammals (monkeys)*** |  |  |  |  |  |  |
| *Hylobates albibarbis* | 1 | 1 | 1 | 1 | 1 | 1 |
| *Macaca fascicularis* | 7 | 7 | 7 | 7 | 7 | 7 |
| *Macaca nemestrina* | 1 | 1 | 1 | 1 | 1 | 1 |
| *Pongo pygmaeus* | 1 | 1 | 1 | 1 | 1 | 1 |
